# (Methanol-κ*O*)(methano­lato-κ*O*)oxido[*N*-(2-oxidobenzyl­idene)phenyl­alaninato-κ^3^ *O*,*N*,*O*′]vanadium(V)

**DOI:** 10.1107/S1600536811003163

**Published:** 2011-01-29

**Authors:** Lin Bian, Lianzhi Li

**Affiliations:** a School of Chemistry and Chemical Engineering, Liaocheng University, Shandong 252059, People’s Republic of China

## Abstract

In the title complex, [V(C_16_H_13_NO_3_)(CH_3_O)O(CH_3_OH)], the V^V^ atom is six-coordinated by a tridentate ligand derived from the condensation of salicyl­aldehyde and l-phenyl­alanine, a vanadyl O atom, a methano­late O atom and a methanol O atom, forming a distorted octa­hedral coordination geometry. In the crystal, inter­molecular O—H⋯O and C—H⋯O hydrogen bonds result in a two-dimensional structure parallel to (001).

## Related literature

For general background to the coordination chemistry of vanadium, see: Diego *et al.* (2003 ▶); Kenji *et al.* (2000 ▶); Thompson *et al.* (1999 ▶); Thompson & Orvig (2006 ▶); Wikksky *et al.* (2001 ▶).
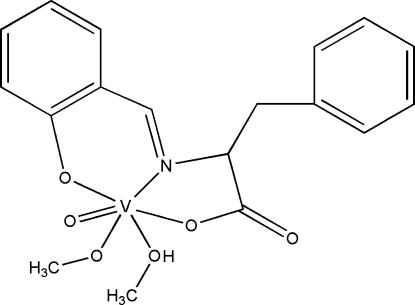


## Experimental

### 

#### Crystal data

[V(C_16_H_13_NO_3_)(CH_3_O)O(CH_4_O)]
*M* *_r_* = 397.29
Orthorhombic, 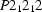

*a* = 14.3095 (15) Å
*b* = 18.782 (2) Å
*c* = 6.6986 (7) Å
*V* = 1800.3 (3) Å^3^
*Z* = 4
Mo *K*α radiation
μ = 0.59 mm^−1^
*T* = 298 K
0.45 × 0.42 × 0.41 mm

#### Data collection

Bruker SMART 1000 CCD diffractometer
Absorption correction: multi-scan (*SADABS*; Sheldrick, 1996 ▶) *T*_min_ = 0.779, *T*_max_ = 0.795
8134 measured reflections
3177 independent reflections
2293 reflections with *I* > 2σ(*I*)
*R*_int_ = 0.073

#### Refinement

*R*[*F* ^2^ > 2σ(*F* ^2^)] = 0.054
*wR*(*F* ^2^) = 0.129
*S* = 1.03
3177 reflections
241 parameters
H atoms treated by a mixture of independent and constrained refinement
Δρ_max_ = 0.46 e Å^−3^
Δρ_min_ = −0.48 e Å^−3^
Absolute structure: Flack (1983 ▶), 1328 Friedel pairs
Flack parameter: 0.04 (4)

### 

Data collection: *SMART* (Bruker, 2007 ▶); cell refinement: *SAINT* (Bruker, 2007 ▶); data reduction: *SAINT*; program(s) used to solve structure: *SHELXS97* (Sheldrick, 2008 ▶); program(s) used to refine structure: *SHELXL97* (Sheldrick, 2008 ▶); molecular graphics: *SHELXTL* (Sheldrick, 2008 ▶) and *DIAMOND* (Brandenburg, 1999 ▶); software used to prepare material for publication: *SHELXTL*.

## Supplementary Material

Crystal structure: contains datablocks global, I. DOI: 10.1107/S1600536811003163/hy2401sup1.cif

Structure factors: contains datablocks I. DOI: 10.1107/S1600536811003163/hy2401Isup2.hkl

Additional supplementary materials:  crystallographic information; 3D view; checkCIF report

## Figures and Tables

**Table 1:** Selected bond lengths (Å)

V1—O1	1.570 (3)
V1—O2	1.934 (3)
V1—O4	1.831 (3)
V1—O5	2.366 (3)
V1—O6	1.762 (3)
V1—N1	2.086 (4)

**Table 2:** Hydrogen-bond geometry (Å, °)

*D*—H⋯*A*	*D*—H	H⋯*A*	*D*⋯*A*	*D*—H⋯*A*
O5—H19⋯O3^i^	1.00 (5)	1.73 (5)	2.687 (4)	161 (4)
C13—H13⋯O3^ii^	0.93	2.59	3.500 (7)	165

Symmetry codes: (i) 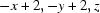; (ii) 
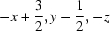
.

**Fig. 1.**
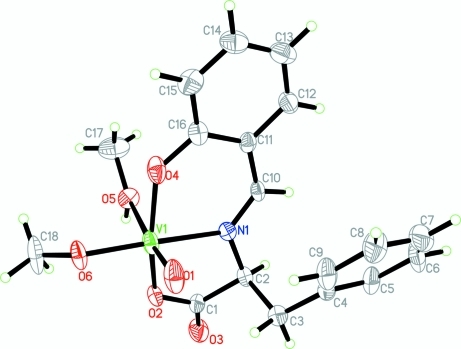
The molecular structure of the title compound, showing 50% probability displacement ellipsoids.

**Fig. 2.**
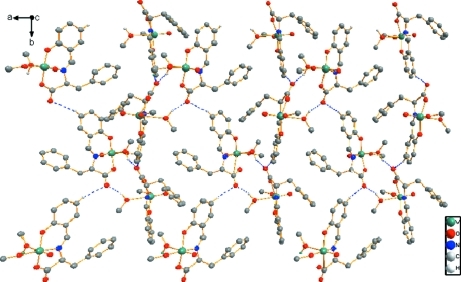
The two-dimensional structure of the title compound, linked by intermolecular hydrogen bonds (dashed lines).

## supplementary crystallographic information

### Comment

The coordination chemistry of vanadium has been receiving increasing interest since it was found that vanadium compounds in various oxidation states have insulin-mimetic properties (Diego *et al.*, 2003; Kenji *et al.*, 2000; Thompson & Orvig, 2006). Compared with other transition metal complexes, less vanadium complexes have been synthesized and characterized (Thompson *et al.*, 1999; Wikksky *et al.*, 2001). We report here the synthesis and crystal structure of an oxovanadium(V) complex with a tetradentate Schiff-base ligand derived from the condensation of salicylaldehyde and *L*-phenylalanine.

As shown in Fig. 1, the V^V^ ion is six-coordinated by the tridentate (O,N,O) donor ligand and three O atoms from an oxido group, a methanolate group and a methanol molecule (Table 1), forming a distorted octahedral geometry. In the complex, O2, N1 and O4 of the tridentate Schiff base ligand and O6 of the methanolate ligand define the equatorial plane and the oxido O1 and the methanol O5 occupy the axial positions. The Schiff base ligand coordinating to the V^V^ atom forms two chelating rings, the five-membered V1—O2—C1—C2—N1 ring and the six-membered V1—N1—C10—C11—C16—O4 ring. The dihedral angle of the two rings is 15.69 (15)°, which increases the stability of the complex.

In the crystal, the intermolecular O—H···O and C—H···O hydrogen bonds (Table 2) result in a two-dimensional structure (Fig. 2).

### Experimental

*L*-Phenylalanine (1 mmol, 0.165 g) and potassium hydroxide (1 mmol, 0.056 g) were dissolved in hot methanol (10 ml) with stirring. The mixture was added successively to a methanol solution (5 ml) of salicylaldehyde (1 mmol, 0.11 ml) with stirring at 323 K for 2 h. Subsequently, an aqueous solution (2 ml) of vanadyl sulfate hydrate (1 mmol, 0.225 g) was added dropwise and stirred for 3 h continuously. The resultant solution was filtered and the filtrate was held at room temperature for several days, whereupon red blocky crystals suitable for X-ray diffraction were obtained.

### Refinement

H atoms on C atoms were placed in calculated positions and refined as riding atoms, with C—H = 0.93–0.98 Å and with *U*_iso_(H) = 1.2(1.5 for methyl)*U*_eq_(C). The hydroxy H atom (H19) of the methanol molecule was located from a difference Fourier map and refined isotropically.

### Crystal data

**Table:**

[V(C_16_H_13_NO_3_)(CH_3_O)O(CH_4_O)]	*F*(000) = 824
*M**_r_* = 397.29	*D*_x_ = 1.466 Mg m^−^^3^
Orthorhombic, *P*2_1_2_1_2	Mo *K*α radiation, λ = 0.71073 Å
Hall symbol: P 2 2ab	Cell parameters from 2512 reflections
*a* = 14.3095 (15) Å	θ = 2.6–23.6°
*b* = 18.782 (2) Å	µ = 0.59 mm^−^^1^
*c* = 6.6986 (7) Å	*T* = 298 K
*V* = 1800.3 (3) Å^3^	Block, red
*Z* = 4	0.45 × 0.42 × 0.41 mm

### Data collection

**Table:**

Bruker SMART 1000 CCD diffractometer	3177 independent reflections
Radiation source: fine-focus sealed tube	2293 reflections with *I* > 2σ(*I*)
graphite	*R*_int_ = 0.073
φ and ω scans	θ_max_ = 25.0°, θ_min_ = 1.8°
Absorption correction: multi-scan (*SADABS*; Sheldrick, 1996)	*h* = −10→17
*T*_min_ = 0.779, *T*_max_ = 0.795	*k* = −22→12
8134 measured reflections	*l* = −7→7

### Refinement

**Table:**

Refinement on *F*^2^	Secondary atom site location: difference Fourier map
Least-squares matrix: full	Hydrogen site location: inferred from neighbouring sites
*R*[*F*^2^ > 2σ(*F*^2^)] = 0.054	H atoms treated by a mixture of independent and constrained refinement
*wR*(*F*^2^) = 0.129	*w* = 1/[σ^2^(*F*_o_^2^) + (0.0594*P*)^2^] where *P* = (*F*_o_^2^ + 2*F*_c_^2^)/3
*S* = 1.03	(Δ/σ)_max_ < 0.001
3177 reflections	Δρ_max_ = 0.46 e Å^−^^3^
241 parameters	Δρ_min_ = −0.48 e Å^−^^3^
0 restraints	Absolute structure: Flack (1983), 1328 Friedel pairs
Primary atom site location: structure-invariant direct methods	Flack parameter: 0.04 (4)

### Fractional atomic coordinates and isotropic or equivalent isotropic displacement parameters (Å^2^)

**Table:**

	*x*	*y*	*z*	*U*_iso_*/*U*_eq_	
V1	0.84142 (5)	0.86795 (4)	0.57437 (11)	0.0419 (3)	
O1	0.7400 (2)	0.8810 (2)	0.6561 (5)	0.0676 (12)	
O2	0.8790 (2)	0.96385 (16)	0.5061 (5)	0.0482 (9)	
O3	0.8863 (2)	1.05118 (18)	0.2846 (6)	0.0613 (10)	
O4	0.8383 (2)	0.77184 (17)	0.5287 (5)	0.0535 (9)	
O5	0.9878 (2)	0.85346 (17)	0.4151 (5)	0.0465 (8)	
O6	0.9045 (2)	0.8721 (2)	0.7999 (4)	0.0549 (9)	
N1	0.7976 (2)	0.87816 (19)	0.2787 (5)	0.0323 (9)	
C1	0.8574 (3)	0.9935 (3)	0.3387 (8)	0.0425 (12)	
C2	0.7896 (3)	0.9517 (2)	0.2140 (7)	0.0369 (11)	
H2	0.8048	0.9560	0.0719	0.044*	
C3	0.6910 (3)	0.9796 (2)	0.2553 (8)	0.0480 (13)	
H3A	0.6811	0.9811	0.3985	0.058*	
H3B	0.6864	1.0280	0.2057	0.058*	
C4	0.6158 (3)	0.9364 (2)	0.1631 (7)	0.0405 (12)	
C5	0.5940 (4)	0.9426 (3)	−0.0351 (8)	0.0579 (15)	
H5	0.6261	0.9753	−0.1138	0.069*	
C6	0.5261 (4)	0.9017 (4)	−0.1182 (10)	0.081 (2)	
H6	0.5114	0.9076	−0.2524	0.097*	
C7	0.4796 (4)	0.8527 (4)	−0.0100 (13)	0.082 (2)	
H7	0.4332	0.8248	−0.0679	0.099*	
C8	0.5020 (4)	0.8451 (3)	0.1849 (12)	0.0731 (19)	
H8	0.4712	0.8109	0.2609	0.088*	
C9	0.5687 (4)	0.8864 (3)	0.2722 (9)	0.0613 (16)	
H9	0.5823	0.8805	0.4069	0.074*	
C10	0.7750 (3)	0.8287 (2)	0.1591 (7)	0.0338 (10)	
H10	0.7585	0.8417	0.0298	0.041*	
C11	0.7727 (3)	0.7552 (2)	0.2063 (7)	0.0363 (11)	
C12	0.7375 (3)	0.7080 (3)	0.0650 (9)	0.0502 (12)	
H12	0.7199	0.7248	−0.0600	0.060*	
C13	0.7286 (3)	0.6378 (3)	0.1077 (9)	0.0607 (15)	
H13	0.7044	0.6068	0.0124	0.073*	
C14	0.7550 (4)	0.6124 (3)	0.2902 (10)	0.0632 (16)	
H14	0.7480	0.5643	0.3190	0.076*	
C15	0.7917 (4)	0.6572 (3)	0.4311 (10)	0.0575 (14)	
H15	0.8104	0.6391	0.5540	0.069*	
C16	0.8010 (3)	0.7291 (3)	0.3918 (7)	0.0436 (12)	
C17	1.0399 (4)	0.7920 (3)	0.3821 (11)	0.078 (2)	
H17A	1.0050	0.7513	0.4264	0.117*	
H17B	1.0975	0.7948	0.4551	0.117*	
H17C	1.0530	0.7874	0.2422	0.117*	
C18	0.9966 (5)	0.8865 (4)	0.8526 (10)	0.096 (2)	
H18A	1.0209	0.9233	0.7677	0.143*	
H18B	1.0334	0.8441	0.8372	0.143*	
H18C	0.9989	0.9019	0.9891	0.143*	
H19	1.024 (4)	0.896 (3)	0.376 (8)	0.065 (17)*	

### Atomic displacement parameters (Å^2^)

**Table:**

	*U*^11^	*U*^22^	*U*^33^	*U*^12^	*U*^13^	*U*^23^
V1	0.0372 (4)	0.0665 (5)	0.0222 (4)	−0.0044 (4)	−0.0019 (4)	−0.0005 (4)
O1	0.042 (2)	0.132 (4)	0.0290 (18)	−0.001 (2)	0.0042 (16)	−0.004 (2)
O2	0.050 (2)	0.057 (2)	0.038 (2)	0.0011 (17)	−0.0172 (16)	−0.0117 (15)
O3	0.068 (2)	0.048 (2)	0.068 (3)	−0.0181 (19)	−0.021 (2)	0.0047 (19)
O4	0.060 (2)	0.062 (2)	0.039 (2)	−0.0178 (18)	−0.0140 (19)	0.0158 (16)
O5	0.0393 (18)	0.051 (2)	0.050 (2)	−0.0013 (16)	0.0070 (18)	0.0033 (19)
O6	0.050 (2)	0.089 (3)	0.0254 (17)	−0.008 (2)	−0.0088 (16)	−0.0017 (19)
N1	0.0256 (19)	0.041 (2)	0.030 (2)	−0.0024 (17)	0.0054 (15)	−0.0029 (18)
C1	0.035 (3)	0.050 (3)	0.043 (3)	0.005 (2)	−0.011 (2)	−0.004 (2)
C2	0.036 (3)	0.044 (3)	0.031 (3)	−0.006 (2)	−0.005 (2)	−0.001 (2)
C3	0.045 (3)	0.049 (3)	0.049 (3)	0.001 (2)	−0.005 (3)	−0.005 (2)
C4	0.034 (3)	0.046 (3)	0.042 (3)	0.002 (2)	−0.005 (2)	−0.007 (2)
C5	0.036 (3)	0.088 (4)	0.049 (4)	−0.001 (3)	−0.010 (3)	0.003 (3)
C6	0.053 (4)	0.135 (6)	0.055 (4)	0.002 (4)	−0.024 (3)	−0.025 (4)
C7	0.038 (3)	0.088 (5)	0.121 (7)	−0.007 (3)	−0.009 (4)	−0.038 (4)
C8	0.035 (3)	0.093 (5)	0.091 (5)	−0.011 (3)	−0.001 (4)	0.008 (4)
C9	0.041 (3)	0.097 (5)	0.047 (3)	−0.013 (3)	−0.004 (3)	0.007 (3)
C10	0.027 (2)	0.051 (3)	0.023 (2)	0.000 (2)	−0.0017 (19)	−0.003 (2)
C11	0.028 (3)	0.048 (3)	0.032 (3)	0.002 (2)	0.000 (2)	−0.001 (2)
C12	0.045 (3)	0.056 (3)	0.049 (3)	0.002 (2)	−0.006 (3)	−0.012 (3)
C13	0.050 (3)	0.051 (3)	0.081 (5)	−0.005 (3)	0.006 (3)	−0.017 (4)
C14	0.070 (4)	0.042 (3)	0.077 (5)	−0.014 (3)	0.004 (4)	0.004 (3)
C15	0.058 (3)	0.054 (3)	0.061 (4)	−0.008 (3)	0.005 (3)	0.019 (3)
C16	0.037 (3)	0.056 (3)	0.039 (3)	−0.007 (2)	0.002 (2)	0.003 (2)
C17	0.067 (4)	0.063 (4)	0.105 (6)	0.005 (3)	0.032 (4)	−0.003 (4)
C18	0.078 (5)	0.165 (7)	0.044 (4)	−0.014 (4)	−0.032 (4)	0.001 (4)

### Geometric parameters (Å, °)

**Table:**

V1—O1	1.570 (3)	C6—H6	0.9300
V1—O2	1.934 (3)	C7—C8	1.352 (9)
V1—O4	1.831 (3)	C7—H7	0.9300
V1—O5	2.366 (3)	C8—C9	1.361 (8)
V1—O6	1.762 (3)	C8—H8	0.9300
V1—N1	2.086 (4)	C9—H9	0.9300
O2—C1	1.289 (5)	C10—C11	1.417 (6)
O3—C1	1.215 (6)	C10—H10	0.9300
O4—C16	1.331 (5)	C11—C12	1.390 (6)
O5—C17	1.392 (6)	C11—C16	1.395 (6)
O5—H19	1.00 (5)	C12—C13	1.356 (6)
O6—C18	1.390 (6)	C12—H12	0.9300
N1—C10	1.269 (5)	C13—C14	1.365 (8)
N1—C2	1.451 (5)	C13—H13	0.9300
C1—C2	1.503 (6)	C14—C15	1.369 (8)
C2—C3	1.531 (6)	C14—H14	0.9300
C2—H2	0.9800	C15—C16	1.382 (6)
C3—C4	1.483 (6)	C15—H15	0.9300
C3—H3A	0.9700	C17—H17A	0.9600
C3—H3B	0.9700	C17—H17B	0.9600
C4—C9	1.368 (7)	C17—H17C	0.9600
C4—C5	1.369 (7)	C18—H18A	0.9600
C5—C6	1.359 (8)	C18—H18B	0.9600
C5—H5	0.9300	C18—H18C	0.9600
C6—C7	1.347 (9)		
			
O1—V1—O6	99.65 (17)	C7—C6—C5	121.3 (7)
O1—V1—O4	100.91 (19)	C7—C6—H6	119.4
O6—V1—O4	101.54 (16)	C5—C6—H6	119.4
O1—V1—O2	101.17 (18)	C6—C7—C8	118.3 (6)
O6—V1—O2	91.07 (15)	C6—C7—H7	120.8
O4—V1—O2	152.27 (15)	C8—C7—H7	120.8
O1—V1—N1	92.22 (16)	C7—C8—C9	121.4 (6)
O6—V1—N1	164.59 (15)	C7—C8—H8	119.3
O4—V1—N1	85.67 (14)	C9—C8—H8	119.3
O2—V1—N1	76.92 (14)	C8—C9—C4	120.5 (6)
O1—V1—O5	173.31 (16)	C8—C9—H9	119.8
O6—V1—O5	86.45 (14)	C4—C9—H9	119.8
O4—V1—O5	80.40 (14)	N1—C10—C11	125.3 (4)
O2—V1—O5	75.80 (13)	N1—C10—H10	117.3
N1—V1—O5	81.31 (12)	C11—C10—H10	117.3
C1—O2—V1	122.7 (3)	C12—C11—C16	119.2 (5)
C16—O4—V1	136.1 (3)	C12—C11—C10	118.5 (4)
C17—O5—V1	129.8 (3)	C16—C11—C10	122.3 (4)
C17—O5—H19	110 (3)	C13—C12—C11	120.7 (5)
V1—O5—H19	119 (3)	C13—C12—H12	119.7
C18—O6—V1	135.4 (3)	C11—C12—H12	119.7
C10—N1—C2	119.2 (4)	C12—C13—C14	120.1 (5)
C10—N1—V1	127.5 (3)	C12—C13—H13	119.9
C2—N1—V1	113.2 (3)	C14—C13—H13	119.9
O3—C1—O2	124.3 (5)	C13—C14—C15	120.7 (5)
O3—C1—C2	121.3 (4)	C13—C14—H14	119.7
O2—C1—C2	114.4 (4)	C15—C14—H14	119.7
N1—C2—C1	106.3 (4)	C14—C15—C16	120.3 (6)
N1—C2—C3	110.2 (4)	C14—C15—H15	119.8
C1—C2—C3	108.4 (4)	C16—C15—H15	119.8
N1—C2—H2	110.6	O4—C16—C15	119.8 (5)
C1—C2—H2	110.6	O4—C16—C11	121.2 (4)
C3—C2—H2	110.6	C15—C16—C11	119.0 (5)
C4—C3—C2	113.9 (4)	O5—C17—H17A	109.5
C4—C3—H3A	108.8	O5—C17—H17B	109.5
C2—C3—H3A	108.8	H17A—C17—H17B	109.5
C4—C3—H3B	108.8	O5—C17—H17C	109.5
C2—C3—H3B	108.8	H17A—C17—H17C	109.5
H3A—C3—H3B	107.7	H17B—C17—H17C	109.5
C9—C4—C5	117.7 (5)	O6—C18—H18A	109.5
C9—C4—C3	120.8 (5)	O6—C18—H18B	109.5
C5—C4—C3	121.5 (5)	H18A—C18—H18B	109.5
C6—C5—C4	120.8 (6)	O6—C18—H18C	109.5
C6—C5—H5	119.6	H18A—C18—H18C	109.5
C4—C5—H5	119.6	H18B—C18—H18C	109.5
			
O1—V1—O2—C1	−83.7 (4)	V1—N1—C2—C3	−90.1 (4)
O6—V1—O2—C1	176.2 (4)	O3—C1—C2—N1	158.9 (4)
O4—V1—O2—C1	58.5 (5)	O2—C1—C2—N1	−23.0 (5)
N1—V1—O2—C1	6.0 (3)	O3—C1—C2—C3	−82.7 (6)
O5—V1—O2—C1	90.1 (3)	O2—C1—C2—C3	95.4 (5)
O1—V1—O4—C16	75.3 (5)	N1—C2—C3—C4	−56.0 (6)
O6—V1—O4—C16	177.7 (4)	C1—C2—C3—C4	−171.9 (4)
O2—V1—O4—C16	−66.9 (6)	C2—C3—C4—C9	97.9 (6)
N1—V1—O4—C16	−16.1 (4)	C2—C3—C4—C5	−79.1 (6)
O5—V1—O4—C16	−98.0 (5)	C9—C4—C5—C6	1.8 (8)
O6—V1—O5—C17	85.4 (5)	C3—C4—C5—C6	178.9 (5)
O4—V1—O5—C17	−16.9 (5)	C4—C5—C6—C7	−1.5 (9)
O2—V1—O5—C17	177.4 (5)	C5—C6—C7—C8	0.1 (10)
N1—V1—O5—C17	−104.0 (5)	C6—C7—C8—C9	1.1 (10)
O1—V1—O6—C18	−157.1 (6)	C7—C8—C9—C4	−0.8 (9)
O4—V1—O6—C18	99.6 (6)	C5—C4—C9—C8	−0.6 (8)
O2—V1—O6—C18	−55.6 (6)	C3—C4—C9—C8	−177.7 (5)
N1—V1—O6—C18	−17.2 (10)	C2—N1—C10—C11	−175.2 (4)
O5—V1—O6—C18	20.1 (6)	V1—N1—C10—C11	1.8 (6)
O1—V1—N1—C10	−95.5 (4)	N1—C10—C11—C12	173.7 (4)
O6—V1—N1—C10	124.0 (6)	N1—C10—C11—C16	−3.7 (7)
O4—V1—N1—C10	5.3 (3)	C16—C11—C12—C13	1.5 (7)
O2—V1—N1—C10	163.6 (4)	C10—C11—C12—C13	−175.9 (4)
O5—V1—N1—C10	86.2 (4)	C11—C12—C13—C14	−0.6 (8)
O1—V1—N1—C2	81.7 (3)	C12—C13—C14—C15	−0.7 (8)
O6—V1—N1—C2	−58.8 (7)	C13—C14—C15—C16	1.1 (8)
O4—V1—N1—C2	−177.5 (3)	V1—O4—C16—C15	−162.9 (4)
O2—V1—N1—C2	−19.3 (3)	V1—O4—C16—C11	18.6 (7)
O5—V1—N1—C2	−96.6 (3)	C14—C15—C16—O4	−178.8 (5)
V1—O2—C1—O3	−173.7 (4)	C14—C15—C16—C11	−0.2 (8)
V1—O2—C1—C2	8.3 (5)	C12—C11—C16—O4	177.5 (4)
C10—N1—C2—C1	−155.4 (4)	C10—C11—C16—O4	−5.2 (7)
V1—N1—C2—C1	27.2 (4)	C12—C11—C16—C15	−1.1 (7)
C10—N1—C2—C3	87.4 (5)	C10—C11—C16—C15	176.2 (4)

### Hydrogen-bond geometry (Å, °)

**Table:**

*D*—H···*A*	*D*—H	H···*A*	*D*···*A*	*D*—H···*A*
O5—H19···O3^i^	1.00 (5)	1.73 (5)	2.687 (4)	161 (4)
C13—H13···O3^ii^	0.93	2.59	3.500 (7)	165

Symmetry codes: (i) −*x*+2, −*y*+2, *z*; (ii) −*x*+3/2, *y*−1/2, −*z*.
